# Impact of Induction Furnace Steel Slag as Replacement for Fired Clay Brick Aggregate on Flexural and Durability Performances of RC Beams

**DOI:** 10.3390/ma14216268

**Published:** 2021-10-21

**Authors:** Md Jihad Miah, Md. Kawsar Ali, Ye Li, Adewumi John Babafemi, Suvash Chandra Paul

**Affiliations:** 1 Department of Civil Engineering, University of Asia Pacific, 74/A, Green Road, Farmgate, Dhaka 1205, Bangladesh; jihad.miah@uap-bd.edu (M.J.M.); kawsar.uap@gmail.com (M.K.A.); 2 School of Civil and Environmental Engineering, Harbin Institute of Technology, Shenzhen 518055, China; liye@hit.edu.cn; 3 Department of Civil Engineering, Stellenbosch University, Private Bag X1, Matieland, Stellenbosch 7602, South Africa; ajbabafemi@sun.ac.za; 4 Department of Civil Engineering, International University of Business Agriculture and Technology, Dhaka 1230, Bangladesh

**Keywords:** induction furnace steel slag, fired clay brick, concrete, flexural strength, durability

## Abstract

This research investigates the flexural and durability performances of reinforced concrete (RC) beams made with induction furnace steel slag aggregate (IFSSA) as a replacement for fired clay brick aggregate (FCBA). To achieve this, 27 RC beams (length: 750 mm, width: 125 mm, height: 200 mm) were made with FCBA replaced by IFSSA at nine replacement levels of 0%, 10%, 20%, 30%, 40%, 50%, 60%, 80%, and 100% (by volume). Flexural tests of RC beams were conducted by a four-point loading test, where the deflection behavior of the beams was monitored through three linear variable displacement transducers (LVDT). The compressive strength and durability properties (i.e., porosity, resistance to chloride ion penetration, and capillary water absorption) were assessed using the same batch of concrete mix used to cast RC beams. The experimental results have shown that the flexural load of RC beams made with IFSSA was significantly higher than the control beam (100% FCBA). The increment of the flexural load was proportional to the content of IFSSA, with an increase of 27% for the beam made with 80% IFSSA than the control beam. The compressive strength of concrete increased by 56% and 61% for the concrete made with 80% and 100% IFSSA, respectively, than the control concrete, which is in good agreement with the flexural load of RC beams. Furthermore, the porosity, resistance to chloride ion penetration, and capillary water absorption were inversely proportional to the increase in the content of IFSSA. For instance, porosity, chloride penetration, and water absorption decreased by 43%, 54%, and 68%, respectively, when IFSSA entirely replaced FCBA. This decreasing percentage of durability properties is in agreement with the flexural load of RC beams. A good linear relationship of porosity with chloride penetration resistance and capillary water absorption was observed.

## 1. Introduction

Globally, the consumption of concrete, being second to water, is increasing every year due to the growing demand for large structures in the construction sector. It is estimated that by 2030, global cement production, a constituent of concrete, may reach around 4.8 billion metric tons [1]. A significant portion of this amount of cement will be used in the production of new concrete. Reports have also shown that the estimated CO_2_ emission (e/m^3^) from one cubic meter of concrete mix made with a water to cement ratio (w/c) of 0.5 is about 347–351 kg [2]. Like cement, a huge number of natural aggregates are also being used in concrete as one of the main constituents as the volumetric stability of concrete is achieved from aggregates. Usually, these aggregates are collected from quarries and water channels. However, natural aggregates are limited in nature and even scarce in many countries. In some countries, coarse aggregates are collected from fired clay brick. However, bricks are fired at very high temperatures, demanding high energy consumption and emitting carbon in the process. Moreover, the collection and recycling of fired brick aggregate are energy-intensive. Depending on the recycling process, such as the conventional mechanical process (grinding by crusher machine) or microwave heating process (heating plus griding), the energy consumption during the recycling of recycled concrete aggregates (RCA) may vary 3–4 times more from one method to another [3].

Furthermore, brick aggregates have higher water absorption and lower strength than the most widely used gravel/stone aggregate, hence serving as the major problem in achieving desirable properties of concrete [4,5]. Finding an alternative to natural aggregates with good quality could be the best option for producing new concrete and maintaining the demand for the future. The major challenge for the construction industry is finding out greener ways to produce concrete as its environmental impact is being questioned repeatedly due to the generation of demolition waste and the exploitation of natural resources [6]. Researchers use different types of alternative aggregates to produce new concrete to minimize the dependency on natural resources, and their different mechanical and durability properties are also being scrutinized extensively [7,8,9,10,11]. The successful use of these waste aggregates can meet the demand for alternatives, minimizing the economic cost and the negative impact on the environment. However, storage or dumping of this waste requires space for landfills, which can pollute the air and water if chemical substances are present in the waste.

Additionally, different industries, such as the steel industry, power plants, etc., produce a vast volume of industrial by-products that need proper management methods. The best option could be to reuse them in building materials like concrete. As such, several researchers have reported on the performance of concrete containing induction furnace steel slag aggregate (IFSSA) as one of the potential types of aggregates for concrete production [12,13]. IFSSA is an industrial by-product of steel manufacturing, considered highly calcareous, siliceous, and ferrous [13]. It is a rock-like material with an angular shape and cavernous inside. Its specific gravity is about 3.4, which is 20–30% higher than normal aggregates and has a low crushing value but more porosity [14]. Nevertheless, the quality and composition of IFSSA vary depending upon the type of furnace and melting temperature, desired steel purity, and the operating conditions of the furnace [15].

Generally, the angular shape of IFSSA in concrete reduces the workability. It is reported that the slump of concrete is reduced to 80% when IFSSA replaces 100% natural aggregates. Thus, concrete with IFSSA may require extra water or superplasticizers to obtain the desired workability [15,16]. However, the segregation and bleeding from extra water in the mix must be checked to achieve the concrete’s expected characteristics. As opposed to the workability, the compressive strength of concrete was increased as the IFSSA content increased. A maximum of 4%, 7%, 21%, and 30% higher strengths of concrete were reported where IFSSA replaced the natural aggregates at 15%, 30%, 50%, and 100%, respectively [15]. Recent studies have reported no significant difference in the concrete strength with 50% and 100% steel slag [17]. Similar to the compressive strength, the flexural and tensile behavior of concrete also improved for IFSSA concrete [15]. An extensive literature survey on concrete’s mechanical properties (39 papers for compressive strength, 21 for split tensile strength, and 19 for flexural strength test) with IFSSA was reported by Brand and Roesler [18]. About 70% of the papers reported that the mechanical properties of concrete improved when IFSSA replaced natural aggregates. The enhanced performance of concrete with IFSSA was attributed to its inherent cementitious behavior, higher angularity, and rough surface, densifying the interfacial transition zone (ITZ) between the cement paste and aggregates [19,20].

In cases where lower strength than the control was reported [18], this could be due to the volume expansion and cracking in the matrix caused by the reaction of excess free CaO in the powder form of IFSSA [20]. Additionally, lower workability and possible segregation or poor casting may also contribute to the lower strength of concrete with IFSSA [20]. However, compared with 100% natural aggregate concrete, the requirements of numerous guidelines were met for classification as a good quality of concrete [21]. Similarly, it was found that 100% IFSSA is suitable as coarse aggregate to produce concrete without compromising the compressive and tensile strength [22].

On the other hand, fired clay brick aggregate (FCBA) is commonly used as coarse aggregate in most countries in South Asia, especially in Bangladesh [22,23]. Fired clay bricks are widely made by molding wet clay into the required shape and size and then burning these clay blocks in an open brickfield kiln at around 1000–1200 °C [24,25,26]. The uncontrolled firing process produces different types of bricks, such as first-class, second-class, third-class, and picket bricks [25,26]. Typically, the first-class bricks are manually crushed to make coarse aggregate [27,28].

In Bangladesh, the concrete industry mainly depends on crushed brick aggregate due to the scarcity of natural stone. However, Miah et al. [22] observed that the concrete made with brick aggregate has significantly lower mechanical strength and higher porosity than conventional concrete. SEM analysis of brick aggregate and concrete made with FCBA showed that brick aggregates contain more voids and cracks. Furthermore, more voids were observed at the cement paste and brick aggregate interface, hence the lower mechanical strength and higher porosity of brick aggregate concrete.

The need for coarse aggregates is rising due to booming construction activities around the globe, particularly in developing countries due to the increasing population. Therefore, a shortage of natural aggregates could be expected in regions where they are limited [29]. Thus, the deficiency in the supply of natural aggregate in South Asia, especially in Bangladesh, has raised the necessity to use alternate coarse aggregate in concrete. In this respect, induction furnace steel slag aggregate (IFSSA), a waste material of the steelmaking industry, could serve as an alternative coarse aggregate [28] and could lower the cost of concrete production.

Several results on the fresh and hardened properties of concrete made with induction IFSSA as a replacement for natural stone aggregate have been reported in the literature. In contrast, almost no research is available that deals with replacing FCBA with IFSSA. Hence, investigations of concrete’s flexural and durability (i.e., porosity, resistance to chloride penetration, and water absorption) performances when IFSSA replaces FCBA are also sparse. Therefore, the shortage of research data, insufficient information, and knowledge on the flexural and durability properties of concrete made with IFSSA as a partial and entire replacement for FCBA motivates this research work. The outcome of this research work could be used as a guideline for designers to make new concrete with this aggregate, which can further boost the country’s circular economy.

Within this context, extensive experimental studies were conducted on the mechanical and durability performances of concrete made with nine different replacement percentages (0%, 10%, 20%, 30%, 40%, 50%, 60%, 80%, and 100% by volume) of FCBA by IFSSA. This research aims to investigate the workability, compressive strength, and flexural performance of RC beams and the durability properties (i.e., porosity, resistance to chloride penetration, and water absorption) of concrete.

## 2. Experimental Methodology

### 2.1. Materials Properties and Mix Design

**Figure 1:**
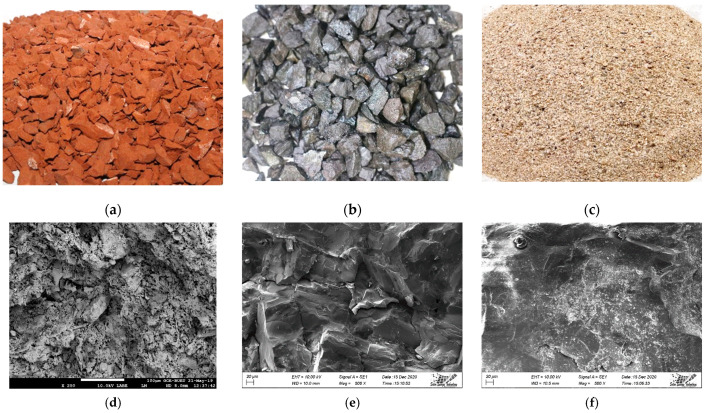
Original image of fired clay brick aggregate-FCBA (**a**), induction furnace steel slag aggregate-IFSSA (**b**), natural sand-NS (**c**), and SEM images of FCBA (**d**), IFSSA (**e**), and NS (**f**), respectively.

The materials used to cast the reinforced concrete (RC) beams for the flexural test and specimens for durability test were binder (CEM II/A-M, 42.5 N [30], consists of 80–94% clinker, 6–20% slag, fly ash, and limestone, and 0–5% gypsum), water, coarse aggregate (fire clay brick aggregate-FCBA and induction furnace steel slag aggregate-IFSSA), and natural sand (NS). The first-class fired clay bricks were collected from a local brickfield to make coarse aggregates, while the waste furnace slag was collected from a steel producing company. The steel slag boulders showed a combination of porous and dense structures [22]. As the content of the denser part was higher in the slag boulders, the slag with the denser structure was used in this study. Both bricks and slag boulders were crushed manually in the laboratory into intermediate sizes to produce coarse aggregate (Figure 1a,b). The natural sand was collected from the local market and used as fine aggregate (Figure 1c).

**Figure 2:**
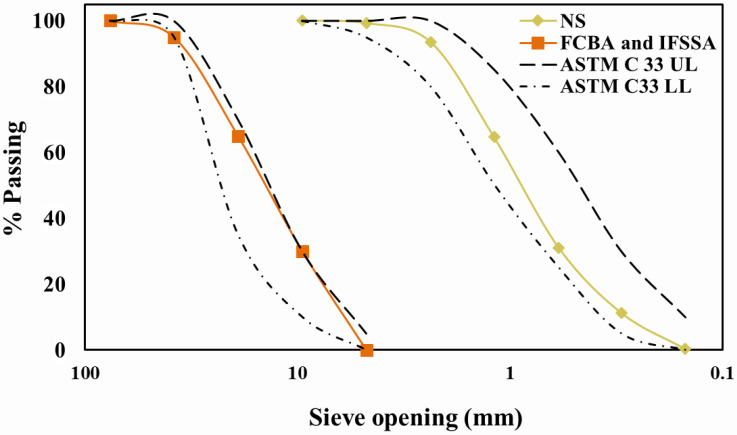
Grain size distribution of FCBA, IFSSA, and NS are plotted with the recommendation provided by the ASTM C33 standard [32].

The microscopic morphology of aggregates (FCBA, IFSSA, and sand) was performed using scanning electron microscopy (SEM, JSM 7600F, JEOL, Tokyo, Japan) and presented in Figure 1d–f. The visual observation and SEM images showed that the IFSSA was denser (less porous) and more highly angular with a rough surface texture compared to FCBA. All aggregates were sieved according to the ASTM C136 standard sieves [31], and the particle size distribution of aggregates is shown in Figure 2. In addition, the gradation curves of the aggregates are presented with the limits recommended by the ASTM C33 standard [32]. It should be noted that a similar gradation for coarse aggregate (FCBA and IFSSA) was used to avoid the effect of gradation on the flexural and durability results of the mixes.

The specific gravity and absorption capacity (ASTM C127-15 [33] and ASTM C128-15 [34]), unit weight (ASTM C29/C29M-17a [35]), and abrasion resistance (ASTM C131/C131M-14 [36]) were tested following ASTM standards for coarse and fine aggregates, as shown in Table 1. The physical properties of aggregates showed that IFSSA has a significantly lower % wear and lower absorption capacity than FCBA due to its dense microstructure (i.e., lower porosity and higher strength) as compared to FCBA. The chemical compositions of FCBA and IFSSA were assessed by Miah et al. [22] using X-ray fluorescence (XRF, LabCenter XRF-1800, Shimadzu, Kyoto, Japan) analysis and are presented in Table 1. No significant change of CaO was found for both FCBA and IFSSA, while FCBA contains more SiO_2_ and MgO than IFSSA. In contrast, the Fe_2_O_3_ of IFSSA was relatively higher than the FCBA, which could be liable for the higher specific gravity and density of IFSSA than FCBA (see Table 1).

The RC beams and durability specimens were cast with nine different concrete mixtures with a w/c of 0.45, and the cement content was 350 kg/m^3^. To investigate the effect of IFSSA on the flexural behavior and durability of RC beams, FCBA was replaced by IFSSA at 0%, 10%, 20%, 30%, 40%, 50%, 60%, 80%, and 100% by volume. As the specific gravity and unit weight of the FCBA and IFSSA differed from others, a volumetric replacement was considered, thus representing a more appropriate approach than weight basis replacement for a comparative study between these two aggregates. The mixture proportion of the concrete is given in Table 2.

### 2.2. Experimental Program and Test Procedures

#### 2.2.1. Flexural Performance of RC Beams

**Figure 3:**
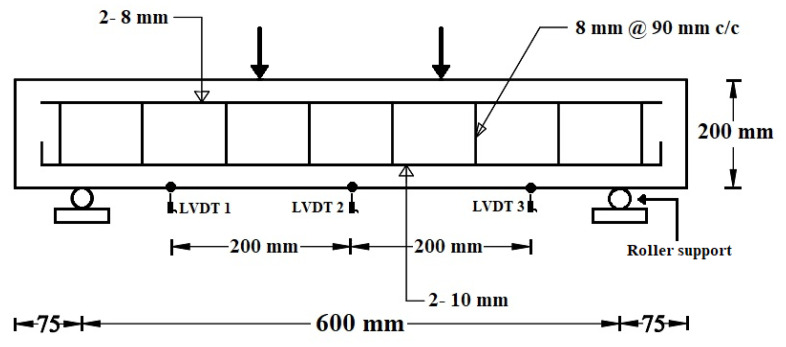
Four-point flexural test of the RC beams made with different percentages of IFSSA.

Twenty-seven RC beams (length: 750 mm, width: 125 mm, height: 200 mm, Figure 3) were cast to investigate the flexural performance of RC beams. Nine replacement percentages (0%, 10%, 20%, 30%, 40%, 50%, 60%, 80%, and 100% by volume) of FCBA by IFSSA were studied, and three beams for each mix were tested to study the influence of IFSSA. The beams were made with 2Ø-10 mm mild steel bars as tension reinforcements at the bottom face and 2Ø-8 mm bars as compression reinforcements at the upper face. Of the 27 beams, three beams made with 100% FCBA are considered as a control. Flexural strength tests of RC beams were conducted at 28 days of curing under four-point bending as per ASTM Standard (ASTM C78-15b [37]) until complete failure of the RC beams with the experimental test setup presented in Figure 3. The RC beams were cured for up to 28 days using wet jute carpets wrapped around the RC beam surfaces. The continuous wetness of the jute carpet to cure the RC beams was ensured by sprinkling water manually 4–6 times a day. During the tests, the deflection of the RC beams was measured through three linear variable displacement transducers (LVDT) at three different locations. One LVDT was positioned at the midpoint (LVDT2), and the other two LVDTs were placed at 200 mm in the left (LVDT 1) and right (LVDT 3) from the midpoint of the RC beam. After the test, the crack patterns and failure behavior were examined. In addition, the slump value of fresh concrete and the compressive strength of hardened concrete at 28 days as per ASTM C39 [38] were assessed using the same batch of concrete mix used to cast RC beams. The cylindrical specimens for compressive strength and durability tests were cured underwater (20 ± 2 °C) until the test day.

#### 2.2.2. Durability of Concrete

The total porosity, chloride penetration resistance, and capillary water absorption tests were performed to investigate the durability properties of RC beams made with different percentages of IFSSA as a replacement for FCBA. The total porosity of concrete mixes was investigated following the recommendation provided by the French standard NF P18-459 [39]. The capillary water absorption tests were performed following AFPC-AFREM [40]. The resistance to chloride penetration test was carried out according to ASTM C1202 [41]. In all tests conducted, a minimum of three specimens was used for each mix. All the specimens for durability tests were made from the same batch of concrete used to make the RC beams, and the durability tests were performed on the day 28 as the RC beams were tested under flexural load.

## 3. Results and Discussion

**Figure 4:**
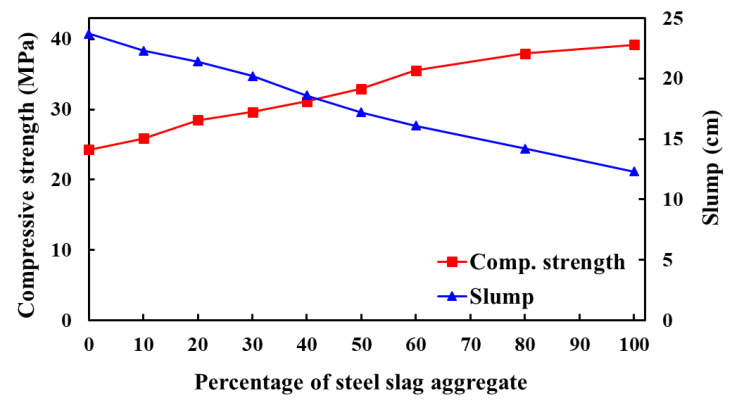
Slump and compressive strength of concrete made with different percentages of IFSSA.

The slump value of freshly mixed concrete and 28 day compressive strength value of different concrete mixtures are shown in Figure 4. Slump value gradually decreased as the percentages of IFSSA increased in the mixture, and this result is in good agreement with the results presented by Miah et al. [22]. This could be attributed to the angular shape of the IFSSA, which may require more water and cement paste for better workability. As opposed to the slump value, the compressive strength of concrete was increased as the IFSSA content increased in the mixes. The compressive strength of concrete increased by 56% and 61% for the concrete made with 80% and 100% IFSSA, respectively, compared to the control concrete, which agrees with the other research [15,22].

### 3.1. Load-Deflection Behavior of RC Beams

**Figure 5:**
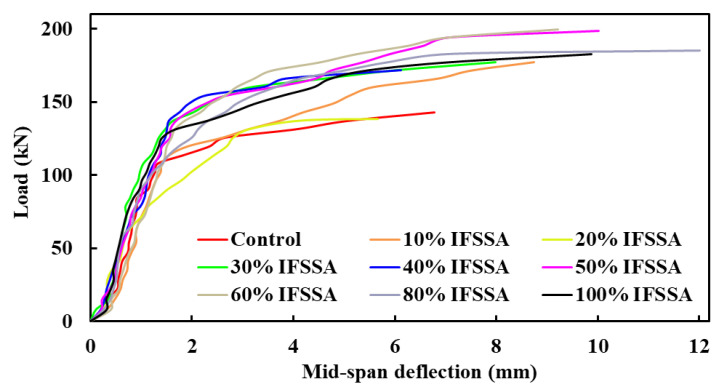
Load-deflection curve of beams made with different percentages of IFSSA.

**Figure 6:**
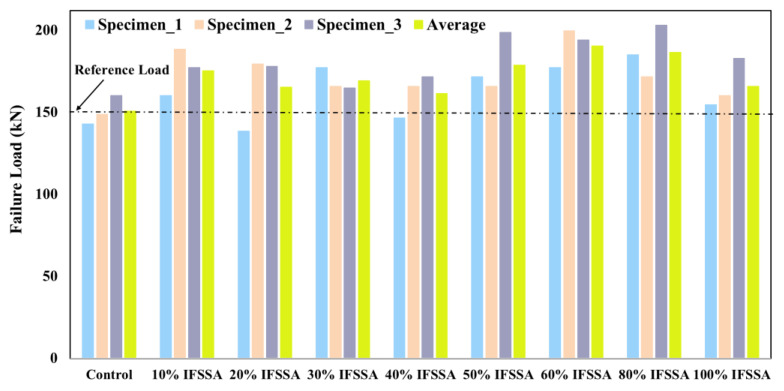
Failure load of RC beams made with different percentages of IFSSA.

The mid-span load-deflection curves of the RC beams are presented in Figure 5. An increase in the stiffness of the RC beams was observed as the content of IFSSA increased in the mixes, with a pronounced effect at a higher content. Initially, the slope of the load-deflection curves was relatively steeper as the cracks and micro-cracks were yet to be formed. However, the slope becomes less steep with the formation of flexural and shear cracks, and finally, the slope tends to zero when the beams nearly fail. As the amount and grade of reinforcement were the same for all beams, the higher stiffness of the beams containing varying contents of IFSSA than 100% FCBA could be linked to the higher modulus of elasticity and higher strength (Figure 4), which delayed and limited the formation of cracks and micro-cracks. As the percentage of IFSSA increases in the specimens, the resistance to the flexural failure of the RC beams increases, and the maximum flexural load was observed for specimens with higher content of IFSSA (Figure 5 and Figure 6). The average failure flexural load of RC beams made with 0%, 10%, 20%, 30%, 40%, 50%, 60%, 80%, and 100% are 150.7 kN, 175.4 kN, 165.3 kN, 169.3 kN, 161.3 kN, 178.7 kN, 190.4 kN, 186.7 kN, and 165.9 kN, respectively (Figure 6). The flexural load increased by 24% for the beam made with 80% IFSSA as compared to the control beam (100% FCBA). However, the maximum failure flexural load among all the beams made with 0% and 80% IFSSA are 160 kN and 203 kN, respectively, about 27% higher than the control beam.

This significantly higher flexural load of RC beams made with IFSSA could be attributed to the higher mechanical strength (Figure 4) and modulus of elasticity (i.e., higher stiffness) of the concrete mix. In turn, the higher mechanical strength could be due to the higher hardness (abrasion resistance: 41.87% for FCBA and 18.89% for IFSSA), dense microstructure (i.e., lower porosity; see Figure 1d,e), excellent surface roughness, and sharp edges of IFSSA compared to FCBA. Furthermore, as the coarse aggregate has the highest contribution to the concrete volume, these better properties of IFSSA may offer a stronger mechanical interlocking around the denser ITZ, resulting in higher mechanical strength and modulus of elasticity, and hence higher flexural load carrying capacity of the RC beams.

Conversely, FCBA particles are weaker (more porous, see Figure 1d), flakier, and lighter than IFSSA, which should be the main reason for the lower strength of concrete, thus offering the RC beams’ low load-carrying capacity. Miah et al. [22] observed that the concrete made with 100% steel slag aggregate had significantly higher mechanical strength than that made with 100% brick aggregate. SEM observations were conducted and found an evident cracking through the brick aggregate and at the ITZ for concrete made with 100% brick aggregate, while no crack of this type and dense ITZ was observed for concrete made with 100% steel slag aggregate.

Furthermore, this behavior could also be linked with the lower porosity and higher resistance to chloride penetration of concrete made with IFSSA than FCBA. It is known that the porosity and pore size distribution of concrete plays an important role in the mechanical strength of concrete, i.e., the mechanical strength of concrete decreases as the porosity increases. Indeed, significantly lower porosity and resistance to chloride penetration were observed for the concrete made with IFSSA. It was more effective for the higher content of IFSSA, which is further discussed in Section 3.3.1 and Section 3.3.2 Moreover, as FCBA has a significantly higher absorption capacity than IFSSA (20.56% for FCBA and 1.2% for IFSSA), the hydration reaction of the cement paste could be affected, leading to porous concrete and lower strength.

Miah et al. [22] reported that brick aggregate’s higher water absorption capacity could create a weak bond between cement paste and aggregate due to absorbed water from surrounding cement paste. This will increase the amount of unhydrated cement in the concrete mix, leading to porous concrete and lower mechanical strength. Therefore, any replacement level of FCBA by IFSSA up to 100% replacement could be recommended to produce concrete and its applications in structures/infrastructures since it would provide better mechanical strength to the RC beams. This also reduces carbon footprint (as the production of FCBA produces a lot of CO_2_) and negative environmental effects (as it is an industrial by-product and produced enormously, thus could create dumping problem), thus supporting an economic and energy-saving sustainable construction material.

Figure 6 shows a slightly lower flexural load for the RC beams with 100% IFSSA than 80% IFSSA. This is inconsistent with the compressive strength results (Figure 4), where strength was maximum at 100% IFSSA content. However, the flexural load of RC beams with 100% IFSSA is higher than with 100% FCBA. This reduction of flexural load could be due to the significantly lower workability (Figure 4) and poor compaction of RC beams, resulting in higher porosity in the RC beam (not for the cylinders) and lower flexural load.

**Figure 7:**
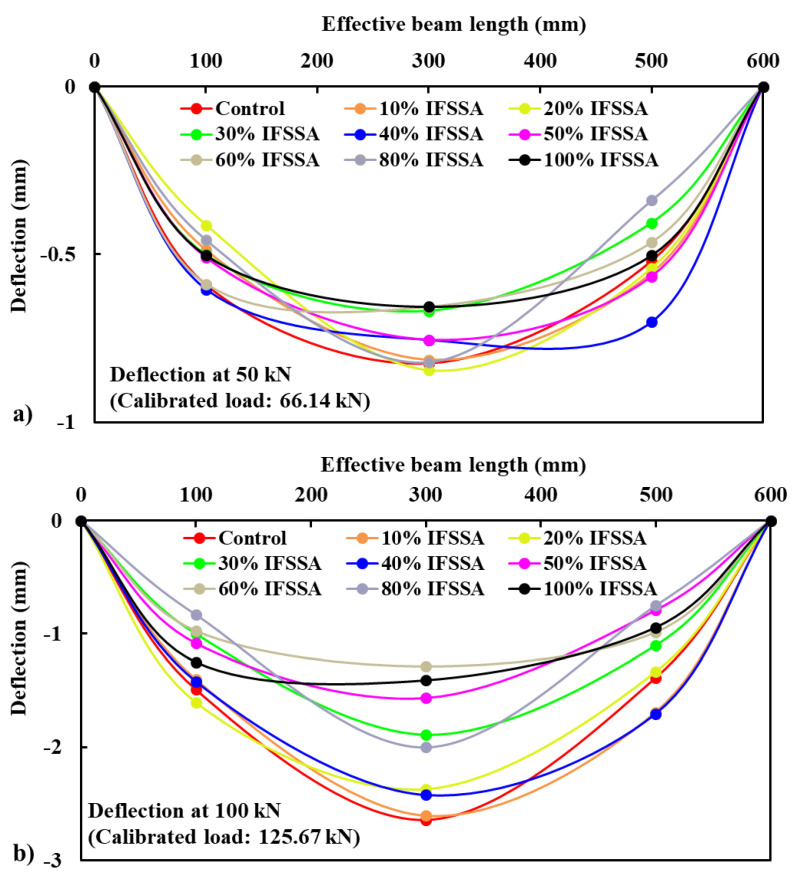
Deformed shape at the fixed flexural load of 50 kN (**a**) and 100 kN (**b**) of beams made with different replacement percentages of FCBA by IFSSA.

The effect of IFSSA on the deformed shape (i.e., sagging of beam under the flexural load) of the RC beams are compared by plotting the deflections of the beams measured at three different points (Figure 3) via LVDTs at fixed flexural loads of 50 kN (actual load after calibration is 66.14 kN) and 100 kN (actual load after calibration is 125.67 kN), and the results are presented in Figure 7. For any given load, the RC beams made with IFSSA provided a less deformed shape than the control beam (100% FCBA), and this behavior was more evident for the higher content of IFSSA. For instance, at 50 kN load, the mid-span deflection of RC beams made with 100% FCBA and 100% IFSSA was 0.83 mm and 0.65 mm, respectively. Similarly, at 100 kN load, it was 2.64 mm and 1.41 mm. These results imply that flexural and shear cracks formed earlier for the control beam than the beam made with IFSSA. Early crack appearance and higher crack number allowed more deflection and deformed toward the flexural loading direction.

**Figure 8:**
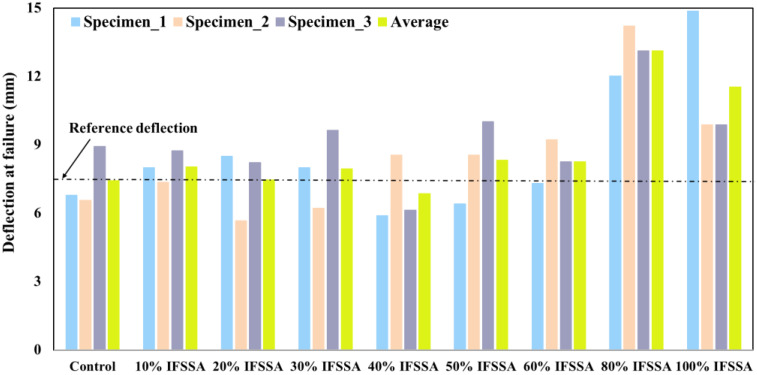
Mid-span deflection at the failure of the RC beams.

In contrast, significantly higher deflection at the failure of the beam was observed for the RC beams made with IFSSA compared to the control beam, and it was more evident for the beams made with a higher content of IFSSA (Figure 8). The maximum deflection of RC beams made with 0%, 80%, and 100% of IFSSA was 8.93 mm, 14.21 mm, and 14.86 mm, respectively, 59% for 80% IFSSA, and 66% for 100% IFSSA, higher than the control beam. This higher ultimate deflection of the RC beams made with IFSSA is due to the flexural higher load-carrying capacity of the beams. This higher deflection of the beams could help to prevent the early collapse of the structure and provide sufficient escape time for the occupants of a building during an extreme external load (e.g., earthquake, Tsunami, and wind load).

### 3.2. Failure Behavior of RC Beams

**Figure 9:**
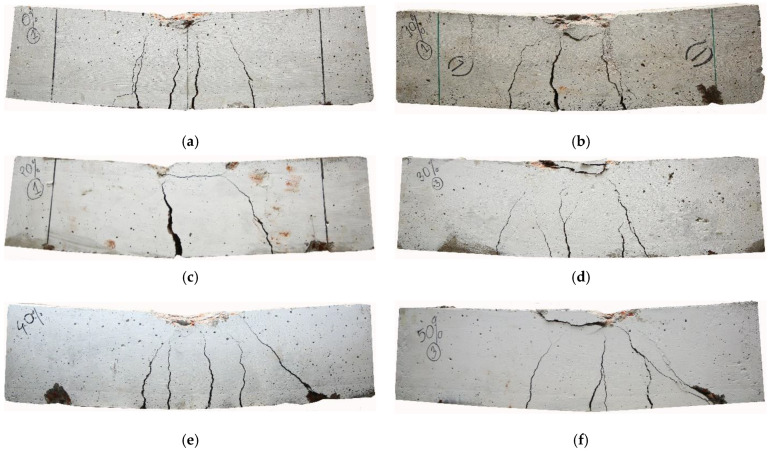

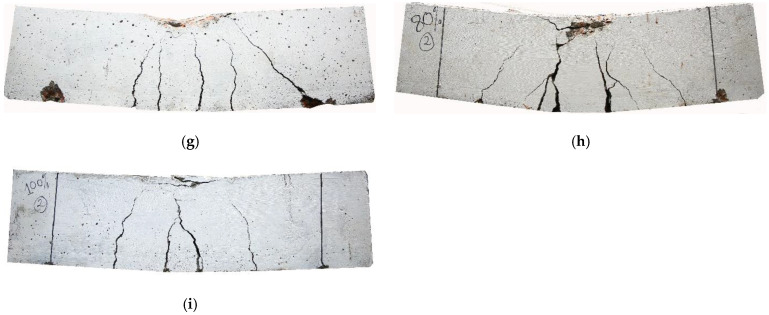
Images of the beams after failure in flexural load: (**a**) Control (0% IFSSA), (**b**) 10% IFSSA, (**c**) 20% IFSSA, (**d**) 30% IFSSA, (**e**) 40% IFSSA, (**f**) 50% IFSSA, (**g**) 60% IFSSA, (**h**) 80% IFSSA, (**i**) 100% IFSSA.

Figure 9 illustrates the images of the RC beams after failure in the flexural load test. The observation during the test shows that as the flexural load increases, flexural cracks appear near the middle span of the beam and then propagate through the height of the RC beam. Afterwards, the diagonal cracks were formed with the continuous increase in the flexural load on the beam. As the load continues, the flexural and diagonal cracks propagate to the compression zone of the beam, and finally, failure occurs. The control beams’ cracking load (i.e., load for the first crack) was much lower than the beams made with IFSSA. For instance, the average cracking load of the beams made with 0% and 100% IFSSA was 61 kN and 103 kN, respectively, which is about 69% higher than the control beam. However, the cracking pattern and number of cracks for beams made with a higher percentage of IFSSA (e.g., 80% IFSSA) differed from the control beam (100% FCBA). The control beam failed with a lower number of flexural and diagonal cracks (Figure 9a). In contrast, the RC beams made with higher percentages of IFSSA exhibited a higher number of flexural and diagonal cracks along the beam’s length (Figure 9g–i) compared to the control beam.

### 3.3. Durability of RC Beam

#### 3.3.1. Porosity

**Figure 10:**
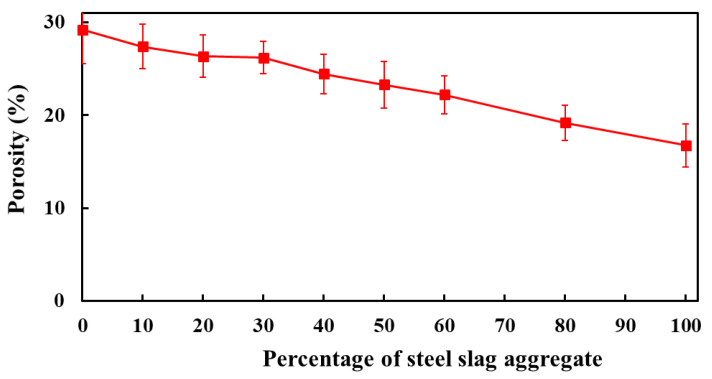
Porosity of concrete made with different percentages of IFSSA.

The total porosity of concrete mixes made with different replacement percentages of FCBA by IFSSA is presented in Figure 10. As the percentage of IFSSA increases, the porosity of the concrete mix decreases, and the maximum decrease was observed when IFSSA entirely replaced FCBA. This decreasing porosity trend with the increasing percentage of IFSSA agrees with the ultimate load-carrying capacity of the RC beams made with IFSSA. Indeed, as concrete’s mechanical strength (compressive strength, Figure 4 and flexural load, Figure 6) increases, the porosity decreases. The average porosity of concrete made with 0%, 10%, 20%, 30%, 40%, 50%, 60%, 80%, and 100% of IFSSA are 29.19%, 27.40%, 26.35%, 26.20%, 24.43%, 23.28%, 22.20%, 19.19%, and 16.74%, respectively. The maximum decrease in total porosity was obtained in the concrete mix made with 100% IFSSA, 43% lower than the control concrete (100% FCBA). This significantly lower porosity of the concrete made with IFSSA than control concrete, especially for the higher content of IFSSA, could be due to the dense microstructure (i.e., stronger due to lower wear of IFSSA than FCBA, Section 2.1) and lower porosity of IFSSA (less permeable and impermeable pores) than FCBA (Figure 1d,e).

It is known that the aggregates occupy about 60–85% of the total volume of concrete, where coarse aggregates are around 40–55% of the concrete volume, which is the highest contributor to the concrete volume. As the IFSSA has less a less porous and dense microstructure than FCBA, and the same amount of cement and sand were used for all the concrete mixes, decreasing porosity with the increasing percentage IFSSA could be directly linked with the lower porosity and dense microstructure of the IFSSA, i.e., as the mortar porosity remains the same for all mixes, the coarse aggregate porosity causes this reduction of the total porosity of concrete. Therefore, as the IFSSA in the mix increases, the porosity decreases. This lower porosity of the concrete offers better mechanical strength to the RC beams (Figure 4, Figure 5 and Figure 6), ensuring better durability and longer service life of the structures/infrastructures made with IFSSA.

#### 3.3.2. Resistance to Chloride Ion Penetration

**Figure 11:**
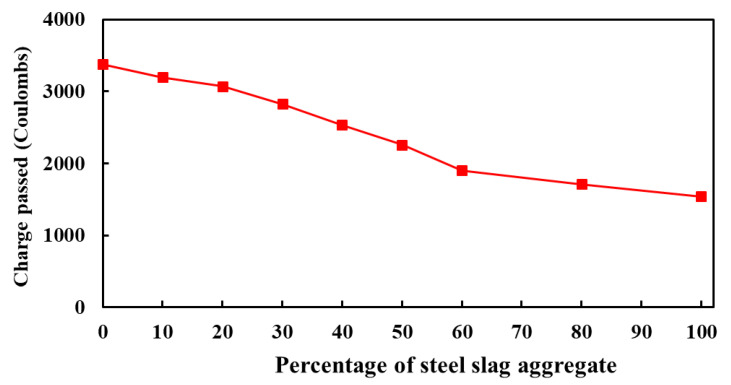
Charge passed into the concrete made with different percentages of IFSSA.

The resistance of concrete mixes to chloride penetration is presented in Figure 11. It is shown that the concrete made with IFSSA has significantly improved the chloride penetration resistance as the transmitted charge in Coulombs (C) was decreased with the increasing percentage of IFSSA. The most remarkable improvement is observed for the concrete made with 100% IFSSA. The average charge passed into concrete mixes made with 0%, 10%, 20%, 30%, 40%, 50%, 60%, 80%, and 100% IFSSA were 3380 C, 3200 C, 3072 C, 2826 C, 2535 C, 2257 C, 1903 C, 1713 C, and 1539 C, respectively.

These results imply that the chloride ion penetration falls in the range from moderate (0% to 50% IFSSA) to low (60% to 100% IFSSA) levels, according to the recommendation provided by the ASTM C1202 [41]. The chloride ion penetration decreased by 54% when IFSSA entirely replaced FCBA. This decreasing trend of chloride ion penetration with the increasing percentage of IFSSA is in good agreement with other test properties (e.g., porosity, compressive strength, and ultimate load-carrying capacity of the RC beams) of concrete mixes. This decreasing trend of chloride ion penetration of concrete mixes made with IFSSA could be attributed to the lower porosity (Figure 10), dense microstructure, and probably lower micro-cracking in the ITZ (because of the higher compressive strength and ultimate load-carrying capacity of the RC beams; see Figure 6 and lower water absorption, discussed in Section 3.3.3) of concrete specimens made with IFSSA than 100% FCBA. This significantly lower chloride ion penetration in the concrete mixes indicates that the inclusion of IFSSA in concrete could provide better protection to steel reinforcement facing corrosion from the harsh environment, resulting in good durability performance of RC beams.

#### 3.3.3. Capillary Water Absorption

**Figure 12:**
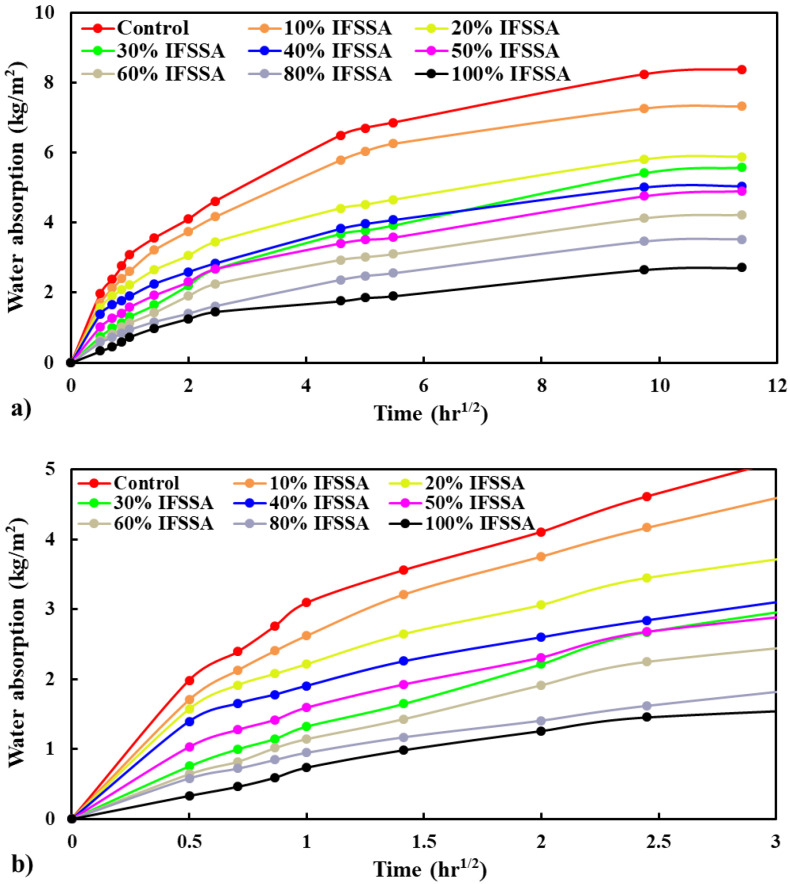
Capillary water absorption (**a**) and detail in data near the origin to represent the slope of the curves (**b**) of concrete specimens made with different percentages of IFSSA.

The average value of the test results is presented in Figure 12a, while the detail of the data from 0 to 6 h is present in Figure 12b. The capillary water absorption is significantly lower for concrete made with IFSSA, and the reduction in water absorption was enormously higher when IFSSA entirely replaced the FCBA (Figure 12a,b). The average capillary water absorption of concrete made with 100% FCBA (control), and 100% IFSSA measured at 130 h was 8.38 kg/m^3^ and 2.71 kg/m^3^, respectively, 68% lower than the control concrete. This reduction of water absorption with the increasing content of IFSSA is in accord with the porosity results (Figure 10). As discussed, this could be due to the significantly lower absorption capacity of IFSSA (20.56% for FCBA and 1.2% for IFSSA), lower porosity, and dense microstructure (i.e., strong ITZ).

As coarse aggregates are the main contributor to the volume of concrete (40–55% of the concrete volume), the water absorption could vary with the coarse aggregate’s porosity and absorption capacity. Since IFSSA has a dense microstructure (less porous and discontinue capillary pore networks, Figure 1e) and significantly lower water absorption capacity than FCBA, this could be the main reason for the lower water absorption of the concrete mix made with IFSSA than FCBA. Therefore, water would be restricted (due to lower permeable pore voids and better ITZ), and slow diffusion of water will occur through the concrete matrix. These results suggested that using IFSSA in concrete increases the resistance against water penetration, enhancing the corrosion resistance of concrete and the durability of RC beams.

#### 3.3.4. Relationship of Durability Properties

**Figure 13:**
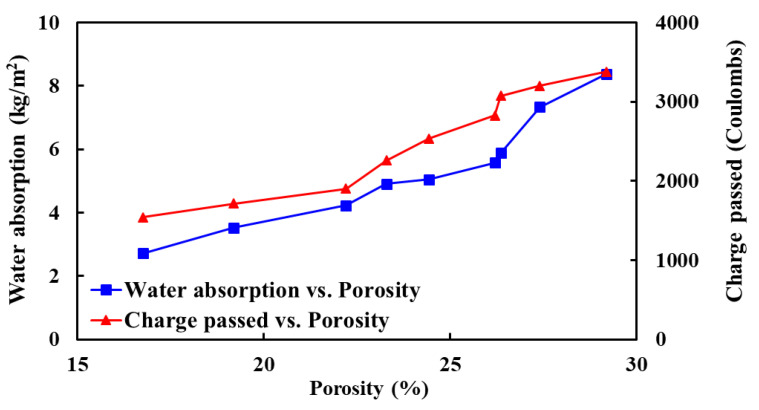
Porosity versus water absorption and charge passed of concrete mixes made with different percentages of IFSSA.

Durability properties play a key role in controlling the longer service life of the concrete structures/infrastructures as concretes are exposed to harsh environments (e.g., exposed to different weathering actions, chemical attack, seawater attack, and abrasion). Therefore, it is essential to have exhaustive knowledge about the durability properties of concrete to guarantee good stability of the structure/infrastructures when exposed to different environmental conditions. Hence, to better understand the durability properties of concrete mixes made with varying percentages of IFSSA, Figure 13 shows the relationship among the porosity, capillary water absorption, and resistance to chloride ion penetration. As expected, the porosity and water absorption have a linear relationship, i.e., as the porosity increases, the capillary water absorption increases. Similarly, the chloride penetration resistance of concrete mixes (i.e., transmitted charge) is related to the total porosity linearly, as shown in Figure 13. These trends imply that the measurement of durability properties is reliable to judge the durability of the RC beams. From these trends, it can be concluded that the inclusion of IFSSA enhances the durability properties of the RC beams better than the beams made with FCBA.

## 4. Conclusions

The present study investigates the role of induction furnace steel slag aggregate (IFSSA) as a partial/entire replacement of fired clay brick aggregate (FCBA) in RC beams’ flexural and durability performance. Nine different replacement levels (0%, 10%, 20%, 30%, 40%, 50%, 60%, 80%, and 100% by volume) of BFCA with IFSSA have been considered. A four-point loading test was conducted for the flexural performance of RC beams, and durability properties (i.e., porosity, chloride penetration resistance, and capillary water absorption) were assessed using the same batch of concrete mix used to cast RC beams. Based on the experimental results, the main findings are summarized as follows:

i. The flexural load increased as the replacement percentage of FCBA by IFSSA increased due to the higher hardness, dense microstructure, surface roughness, and sharp edges of IFSSA and improved ITZ. The flexural load increased by 27% for the beam made with 80% IFSSA compared to the control beam (100% FCBA).
ii. The RC beams made with IFSSA have higher stiffness than the control beam (100% FCBA), probably due to higher modulus of elasticity and higher strength of concrete, delaying and limiting the formation of cracks and micro-cracks. However, the RC beams with IFSSA failed with significantly higher deflection than control beams, 59% for 80% IFSSA and 66% for 100% IFSSA, higher than the control beam.
iii. Porosity, resistance to chloride ion penetration, and capillary water absorption decreased with the increasing replacement of FCBA by IFSSA, which are 43%, 54%, and 68%, respectively, lower when IFSSA entirely replaced FCBA.
iv. Linear relationships between porosity, water absorption, and resistance to chloride penetration of concrete mixes were observed, i.e., as the porosity increases, the capillary water absorption and resistance to chloride penetration of concrete mixes increase.

This study reveals that IFSSA can be used as a complete replacement of FCBA as it provides significantly higher flexural load, higher deflection at failure, and better durability than control concrete. Furthermore, IFSSA provides environmental solutions by solving the dumping problem, reducing the demand for new coarse aggregate, and thereby the carbon footprint, to provide a more economical and sustainable green construction building material as producing bricks by burning also produces a lot of carbon dioxide. Though there are clear advantages under the virgin conditions observed, future research work needs to be carried out to better understand the fire behavior [42,43,44] of concrete structural members made with IFSSA to guarantee good fire protection of structures.

## Figures and Tables

**Table 1:** Physical and chemical properties of aggregates.

	FCBA	IFSSA	Sand
Specific gravity	2.0	3.24	2.62
Unit weight (kg/m^3^)	1140	1810	1565
Abrasion resistance (%)	41.87	18.89	-
Absorption capacity (%)	20.56	1.2	5.86
CaO (%)	4.18	4.94	-
SiO_2_ (%)	60.43	26.18	-
Fe_2_O_3_ (%)	14.27	44.39	-
Al_2_O_3_ (%)	9.96	4.93	-
MgO (%)	1.69	0.46	-
K_2_O (%)	5.23	0.56	-
TiO_2_ (%)	1.81	1.73	-
MnO (%)	0.30	12.9	-
Na_2_O (%)	0.90	0.45	-
ZnO (%)	0.10	2.33	-
SO_3_ (%)	0.57	0.43	-
P_2_O_5_ (%)	0.24	0.08	-
SrO (%)	0.05	0.09	-
ZrO_2_ (%)	0.05	0.11	-

**Table 2:** Mix design of the concrete mixes (kg/m^3^).

Mix ID.	% FCBA	% IFSSA	Cement	Coarse Aggregate		NS	Water
				FCBA	IFSSA		
Control	100	0	350	781	0	817	158
10% IFSSA	90	10	350	703	126	817	158
20% IFSSA	80	20	350	624	253	817	158
30% IFSSA	70	30	350	546	379	817	158
40% IFSSA	60	40	350	468	506	817	158
50% IFSSA	50	50	350	390	632	817	158
60% IFSSA	40	60	350	312	759	817	158
80% IFSSA	20	80	350	156	1012	817	158
100% IFSSA	0	100	350	0	1265	817	158

## Data Availability

The data presented in this study are available on request from the corresponding author.
